# Body weight-based iodinated contrast immersion timing for human fetal postmortem microfocus computed tomography

**DOI:** 10.1093/bjro/tzad006

**Published:** 2023-12-12

**Authors:** Ian C Simcock, Susan C Shelmerdine, John Ciaran Hutchinson, Neil J Sebire, Owen J Arthurs

**Affiliations:** Department of Clinical Radiology, Great Ormond Street Hospital for Children, London WC1N 3JH, United Kingdom; UCL Great Ormond Street Institute of Child Health, Great Ormond Street Hospital for Children, London WC1N 1EH, United Kingdom; NIHR Great Ormond Street Hospital Biomedical Research Centre, London WC1N 1EH, United Kingdom; Department of Clinical Radiology, Great Ormond Street Hospital for Children, London WC1N 3JH, United Kingdom; UCL Great Ormond Street Institute of Child Health, Great Ormond Street Hospital for Children, London WC1N 1EH, United Kingdom; NIHR Great Ormond Street Hospital Biomedical Research Centre, London WC1N 1EH, United Kingdom; NIHR Great Ormond Street Hospital Biomedical Research Centre, London WC1N 1EH, United Kingdom; Department of Histopathology, Great Ormond Street Hospital for Children, London WC1N 3JH, United Kingdom; UCL Great Ormond Street Institute of Child Health, Great Ormond Street Hospital for Children, London WC1N 1EH, United Kingdom; NIHR Great Ormond Street Hospital Biomedical Research Centre, London WC1N 1EH, United Kingdom; Department of Histopathology, Great Ormond Street Hospital for Children, London WC1N 3JH, United Kingdom; Department of Clinical Radiology, Great Ormond Street Hospital for Children, London WC1N 3JH, United Kingdom; UCL Great Ormond Street Institute of Child Health, Great Ormond Street Hospital for Children, London WC1N 1EH, United Kingdom; NIHR Great Ormond Street Hospital Biomedical Research Centre, London WC1N 1EH, United Kingdom

**Keywords:** micro-CT, fetal, potassium tri-iodide, immersion time

## Abstract

**Objectives:**

The aim of this study was to evaluate the length of time required to achieve full iodination using potassium tri-iodide as a contrast agent, prior to human fetal postmortem microfocus computed tomography (micro-CT) imaging.

**Methods:**

Prospective assessment of optimal contrast iodination was conducted across 157 human fetuses (postmortem weight range 2-298 g; gestational age range 12-37 weeks), following micro-CT imaging. Simple linear regression was conducted to analyse which fetal demographic factors could produce the most accurate estimate for optimal iodination time.

**Results:**

Postmortem body weight (*r*^2^ = 0.6435) was better correlated with iodination time than gestational age (*r*^2^ = 0.1384), producing a line of best fit, *y* = [0.0304 × body weight (g)] − 2.2103. This can be simplified for clinical use whereby immersion time (days) = [0.03 × body weight (g)] − 2.2. Using this formula, for example, a 100-g fetus would take 5.2 days to reach optimal contrast enhancement.

**Conclusions:**

The simplified equation can now be used to provide estimation times for fetal contrast preparation time prior to micro-CT imaging and can be used to manage service throughput and parental expectation for return of their fetus.

**Advances in knowledge:**

A simple equation from empirical data can now be used to estimate preparation time for human fetal postmortem micro-CT imaging.

## Introduction

Following pregnancy loss, postmortem investigations can provide additional information in 40-70% of cases, thereby assisting in the grieving process for the family and possibly influencing future pregnancy decisions.^1–3^ However, parental uptake of these investigations is low at 55%, with the invasive nature of conventional autopsy techniques often cited by families as being the main barrier.^1^ Less-invasive options have been shown to be more acceptable to parents,^4^ providing similar diagnostic accuracy in fetuses >20 weeks of gestation.^5–13^ However, many of the standard 1.5-T MRI or CT techniques do not give sufficient image resolution below 20 weeks of gestation.^5^^,^^6^^,^^14–16^

Microfocus computed tomography (micro-CT) is an imaging technique that has been demonstrated to achieve micron-level resolution of early gestation fetuses (<20 weeks).^5^^,^^6^^,^^14–22^ To differentiate internal soft tissue structures with micro-CT, an exogenous contrast agent is required, typically using potassium tri-iodide (I_2_KI).^14–16^^,^^18–29^ The fetus is placed within this solution, and the agent slowly diffuses through the skin and internal organs over the period of several days. Failure to diffuse completely across the fetal structures can lead to missed diagnoses due to the lack of visualization of key organs. Currently, there is debate regarding the optimal contrast immersion time across a range of specimen sizes for satisfactory diagnostic imaging results, ranging from 48 h to 7 days.^16^^,^^21^ This could result in fetuses being imaged at arbitrary time points, sometimes repeatedly until optimum iodination is achieved, and parents and mortuary staff are left without knowing how long to expect an imaging-based autopsy (using micro-CT) to take.

Accurate estimation of the optimal iodine immersion time would allow staffing and scanner usage to be optimized, minimize the risks of incomplete iodination prior to imaging interpretation, and improve service delivery workflow efficiency, whilst also managing parental expectations appropriately.

In this study, we evaluate the relationship between fetal body weight and gestational age to determine how optimal tissue iodination timing can be estimated prior to human fetal postmortem micro-CT.

## Methods

### Patient data collection

Consecutive, unselected fetuses below 300 g of body weight were prospectively included over a 1-year period between October 2019 and October 2020, following parental consent for postmortem micro-CT imaging. Routine consent for imaging was obtained by the clinical team at the referring hospital.

Fetuses >300 g were excluded from micro-CT scanning in our study for 2 reasons: they can be imaged with other postmortem modalities without need for iodine staining (eg, ultrasound or MRI) and our preliminary results have previously indicated that these would require >2 weeks’ immersion, unacceptable to most parents. Further exclusion criteria for this study included fetuses where clinical service requirements delayed the scanning date, or where an anatomical abnormality could affect the iodine perfusion, such as a body wall defect.

### Micro-CT scanning and I_2_KI immersion

**Figure 1.**
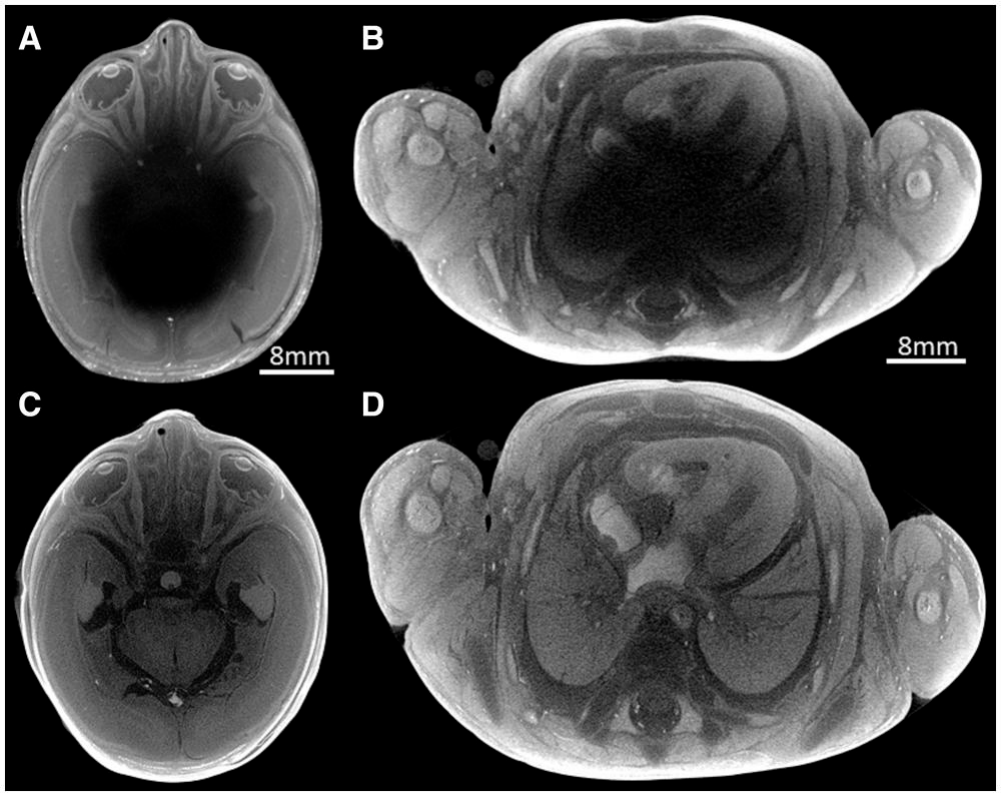
Axial micro-CT example images of a 265-g fetus at 20 weeks of gestation demonstrate incomplete iodination (A and B) after 7 days, but complete iodination (C and D) in the head and chest after 10 days.

Fetuses were immersed in 1 L of 2.5% I_2_KI solution (50 g of I_2_ and 100 g of KI dissolved in 1000 mL H_2_O, followed by 1:1 dilution with 10% formalin) as indicated in previous studies,^17^^,^^20^^,^^21^^,^^26^^,^^29–32^ and stored at room temperature without agitation or light source. The solution was not replenished. The purpose was to create a protocol that would require no specialist equipment and could be easily implemented into clinical practice. Scanning was completed on either XTH225 ST or Med-X micro-CT scanner (Nikon Metrology, Tring, UK), depending on availability. All micro-CT scans were completed as previously published in detail, with a range of imaging parameters depending on the size of fetus being imaged (100-130 kV, 150-250 µA, 250 ms, and 1 FPP).^17^ The entire fetus was included within the x-ray beam to allow rapid assessment (<10 min scan time) of the stage of iodination for all areas of anatomy, repeated imaging every 3 days until full iodination was observed. If insufficiently iodinated (eg, Figure 1), the fetus was replaced within the I_2_KI solution and scanning (after fetal re-immersion in the iodinated solution) was repeated between 24 and 72 h later until complete iodination was observed. Complete iodination was identified when all central anatomical regions were visible (Figure 1).

### Data analysis

Fetal postmortem body weight prior to immersion and gestational age was recorded. Gestational age (rounded to the nearest whole number of weeks) was recorded from the antenatal medical records. Data were recorded using Microsoft Excel version 365 (Microsoft Corp, Redmond WA, USA), and simple linear regression analysis was used to compare parameters.

## Results

### Study population

A total of 194 fetuses were referred for micro-CT scanning between October 2019 and October 2020, of which 157 (80.9%) fetuses were included in the study. We excluded 37 (19.1%) fetuses from further analysis, 36 due to incomplete documentation of immersion times, and 1 fetus had an abdominal wall defect which may have affected the results.

Postmortem body weight range was 2-298 g (median 73.9 g), with 89 (56.7%) at 0-100 g, 38 (24.2%) at 101-200 g, and 30 (19.1%) at 201-300 g. Gestational ages ranged from 12 to 34 weeks (median 18 weeks), with 115 (73.2%) fetuses at 12-19 weeks, 40 (25.5%) fetuses at 20-28 weeks, and 2 (1.3%) fetuses at 29-37 weeks.

**Figure 2.**
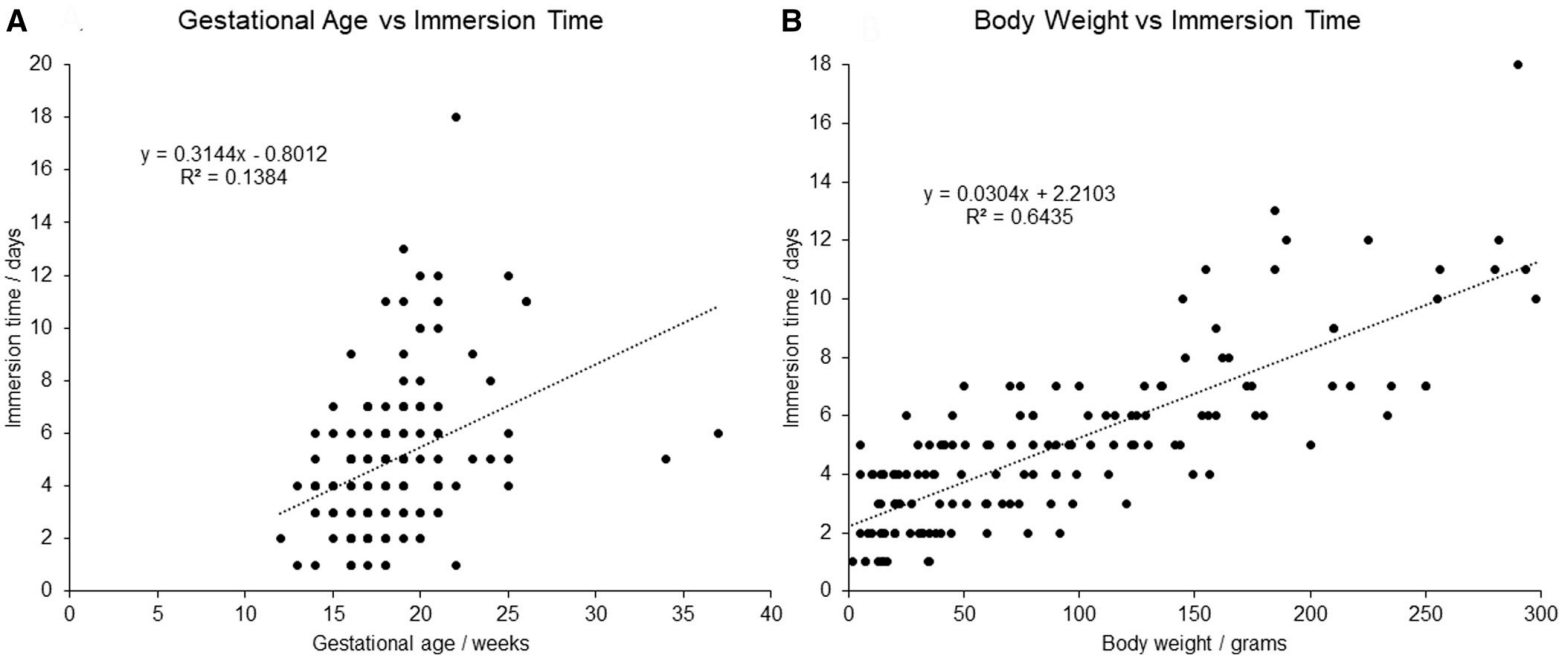
Simple linear regression analysis demonstrates that gestational age was a poor predictor (*r*^2^ = 0.14), and body weight was a good predictor of immersion time (*r*^2^ = 0.64).

Simple linear regression analysis comparing body weight and immersion time resulted in an *r*^2^ value of 0.6435 (Figure 2). This also provided a line of best fit of immersion time = (0.0304 × body weight) − 2.2103, which can be approximated to the equation: immersion time (days) = 0.03 × Weight (g) + 2.2.

Using this formula, a fetus of 100 g would take 5.2 days, 200 g would take 8.2 days, and 300 g would take 11.2 days.

Simple linear regression analysis comparing gestational age and immersion time resulted in an *r*^2^ value of 0.1384, indicating a poor correlation (Figure 2).

Gestational age correlated poorly with body weight in our study (*r*^2^ = 0.1721).

## Discussion

This study has shown that body weight (*r*^2^ = 0.63), rather than gestational age (*r*^2^ = 0.13), has better correlation with the required immersion time in 2.5% I_2_KI solution for optimal contrast enhancement across a range of human fetuses. The simplified formula of Immersion time (days) = 0.03 × weight (g) + 2.2 can be used to estimate the immersion time required to achieve full iodination for fetal postmortem micro-CT.

Whilst other studies have demonstrated proof of principle and diagnostic accuracy of human fetal micro-CT scanning using I_2_KI solution,^14–16^^,^^18–22^^,^^24^^,^^26–29^^,^^32^ this is the largest study of 157 cases evaluating predictors of optimal iodination immersion time for diagnostic interpretation, over a large range of body weights (2-298 g) and gestational ages (mostly 12-26 weeks).

It is not surprising that gestational age is a poor indicator of iodination immersion time, as iodination occurs through simple diffusion at a fixed rate, which is highly likely to be dependent upon size or mass. Other biological specimen investigations^23^^,^^33^ determined that specimen weight was an important variable of iodination time, with larger specimens requiring greater exposure to I_2_KI through longer immersion times.

Different postmortem fetal weights at the same gestational age are accounted for through a number of possibilities. Following *in utero* fetal demise of a fetus, the gestational age at delivery may not reflect gestational age at demise and is therefore often an inaccurate estimate of the size of the fetus. Together with variable duration of autolysis or maceration (*in utero* decomposition), the gestational age at delivery is a poor predictor of structural tissue integrity and thus permeability to exogenous contrast agents.

This study has several limitations. We did not scan on a daily basis and therefore our data approximate “time to full iodination,” and 3-day intervals gave a reproducible accurate estimate. Whilst we understand that maceration may be a factor in the diffusion of iodine through the fetal body, we did not measure this feature as it is rather subjective and hard to control for. Maceration describes changes that occur secondary to intrauterine fetal retention following fetal death as well as tissue autolysis^34^ and may speed up the iodination process due to tissue breakdown and destroying barriers to fluid and thus iodine movement and could account for some of the variation in our dataset. Our population only included fetuses <300 g due to other postmortem imaging techniques demonstrating suitability above this size^5^^,^^6^^,^^8–10^^,^^12^^,^^16^^,^^35–39^ and the time required to iodinate larger specimens in contradiction to parents wishing to have their babies returned quickly. Whilst we recognize that this represents a small weight range, micro-CT has been demonstrated to be clinically useful in smaller fetuses^14–16^^,^^18^^,^^19^^,^^31^ and feel that this establishes a suitable clinical protocol within a defined clinical cohort.

Whilst temperature, agitation, degree of maceration, and concentration of the iodine solution may all affect the iodination time, we aimed to develop a simple clinical protocol using an I_2_KI concentration widely quoted within the literature.^17^^,^^20^^,^^21^^,^^26^^,^^29^^,^^30^^,^^32^

It is also noted that a small study of 3 human fetuses identified buffered solutions of I_2_KI to reduce tissue distortion.^24^ We did not encounter significant tissue distortion in our study that achieved clinically diagnostic imaging in over 150 fetuses, and therefore did not buffer the solution, although this could be incorporated into future protocol developments.

Our data demonstrate that body weight is better correlated with optimal iodine immersion time than gestational age. It should be used to estimate the immersion time required for human fetal postmortem micro-CT cases, allowing better staff and service management and parental expectation of procedure timing during consenting. Greater knowledge in this emerging postmortem imaging technique will enable it to be more easily adopted in new specialist centres, through more standardized clinical protocols, and increase the availability of this technique to more families, providing an increase in investigations following pregnancy loss. This could increase the knowledge available to clinicians and parents alike following a pregnancy loss, an area of medicine that remains under-investigated.

## Conclusion

A simple equation can be used to estimate fetal tissue preparation time for optimal micro-CT imaging. This can now be used to manage service throughput and parental expectation for human fetal postmortem imaging.
